# Origin and evolution of the RIG-I like RNA helicase gene family

**DOI:** 10.1186/1471-2148-9-85

**Published:** 2009-04-28

**Authors:** Jun Zou, Mingxian Chang, Pin Nie, Chris J Secombes

**Affiliations:** 1 Scottish Fish Immunology Research Centre, School of Biological Sciences, University of Aberdeen, Aberdeen, AB24 2TZ, UK; 2 State Key Laboratory of Freshwater Ecology and Biotechnology Institute of Hydrobiology, Chinese Academy of Sciences, 7 South Donghu Road, Wuhan, 430072, PR China

## Abstract

**Background:**

The DExD/H domain containing RNA helicases such as retinoic acid-inducible gene I (RIG-I) and melanoma differentiation-associated gene 5 (MDA5) are key cytosolic pattern recognition receptors (PRRs) for detecting nucleotide pathogen associated molecular patterns (PAMPs) of invading viruses. The RIG-I and MDA5 proteins differentially recognise conserved PAMPs in double stranded or single stranded viral RNA molecules, leading to activation of the interferon system in vertebrates. They share three core protein domains including a RNA helicase domain near the C terminus (HELICc), one or more caspase activation and recruitment domains (CARDs) and an ATP dependent DExD/H domain. The RIG-I/MDA5 directed interferon response is negatively regulated by laboratory of genetics and physiology 2 (LGP2) and is believed to be controlled by the mitochondria antiviral signalling protein (MAVS), a CARD containing protein associated with mitochondria.

**Results:**

The DExD/H containing RNA helicases including RIG-I, MDA5 and LGP2 were analysed *in silico *in a wide spectrum of invertebrate and vertebrate genomes. The gene synteny of MDA5 and LGP2 is well conserved among vertebrates whilst conservation of the gene synteny of RIG-I is less apparent. Invertebrate homologues had a closer phylogenetic relationship with the vertebrate RIG-Is than the MDA5/LGP2 molecules, suggesting the RIG-I homologues may have emerged earlier in evolution, possibly prior to the appearance of vertebrates. Our data suggest that the RIG-I like helicases possibly originated from three distinct genes coding for the core domains including the HELICc, CARD and ATP dependent DExD/H domains through gene fusion and gene/domain duplication. Furthermore, presence of domains similar to a prokaryotic DNA restriction enzyme III domain (Res III), and a zinc finger domain of transcription factor (TF) IIS have been detected by bioinformatic analysis.

**Conclusion:**

The RIG-I/MDA5 viral surveillance system is conserved in vertebrates. The RIG-I like helicase family appears to have evolved from a common ancestor that originated from genes encoding different core functional domains. Diversification of core functional domains might be fundamental to their functional divergence in terms of recognition of different viral PAMPs.

## Background

Pattern recognition receptors (PRRs) are crucial to animal surveillance of pathogen invasion. The PRRs recognise conserved pathogen-associated molecular pattern (PAMP) motifs, including proteins, lipids and nucleotides, resulting in activation of host innate defences [1]. The PRRs comprise three major groups, toll like receptors (TLR), retinoic acid induced RIG-I like receptors and nucleotide oligomerization domain (NOD) containing proteins, sensing PAMPs extracellularly or within the cytoplasmic region.

The RIG-I like receptors are crucial to the RNA virus triggered interferon response. They consist of three members, retinoic acid-inducible gene I (RIG-I, also named DEAD (Asp-Glu-Ala-Asp) box polypeptide 58 (DDX58)) and melanoma differentiation-associated gene 5 (MDA5, also named interferon induced with helicase C domain 1 (IFIH1)), and laboratory of genetics and physiology 2 (LGP2, also named DExH (Asp-Glu-X-His) box polypeptide 58 (DHX58)), which share a common functional RNA helicase domain near the C terminus (HELICc) specifically binding to the RNA molecules with viral origin [2-4]. Two tandem arranged caspase activation and recruitment domains (CARDs) involved in protein-protein interactions are present at the N terminal region of the RIG-I and MDA5 proteins but not LGP2, triggering the interferon response via activation of interferon regulatory factor 3 and NFkB [3,5]. Another distinct core domain is the ATP dependent DExD/H domain containing a conserved motif Asp-Glu-X-Asp/His (DExD/H) which is involved in ATP-dependent RNA or DNA unwinding. RIG-I/MDA5 directed interferon signalling is now known to be controlled by the mitochondria antiviral signalling protein (MAVS), a CARD containing protein associated with mitochondria, and negatively regulated by LGP2 which lacks a CARD domain [4,6,7]. LGP2 has been shown to interfere with the binding process of RIG-I/MDA5 to viral RNAs [8].

Both RIG-I and MDA5 appear to have overlapping binding properties with viral PAMPs and share similar signalling pathways leading to activation of the interferon system. However, evidence of differential recognition of viral PAMPs by RIG-I has begun to emerge recently. It seems that MDA5 preferentially binds long, capped di- or mono-5' phosphate double stranded (ds) RNAs whilst RIG-I has high binding affinity with short dsRNAs or 5' ppp uncapped single stranded (ss) RNAs [9-11]. Interestingly, neither RIG-I nor MDA5 has a classic RNA binding motif. A zinc-binding domain located at the C terminal region (802–925 aa) of human has been shown to specifically bind to viral derived 5'ppp RNA [12,13]. RIG-I and MDA5 respond differently to infection with various viral strains, with RIG-I sensitive to paramyxoviruses, orthomyxoviruses, and the rhabdovirus *vesicular stomatitis *virus whilst MDA5 reacts to picornaviruses [11,14]. Some viral proteins, such as the V protein of paramyxoviruses, interact with MDA5, a mechanism possibly used by viruses as a means to escape host surveillance.

Whilst most studies are focused on the RIG-I like PRRs in mammals, little is known about such molecules in other living organisms. A recent study surveying the purple sea urchin genome has revealed multiple putative RIG-I like homologues that appear to be present in invertebrates [15]. More recently, it has been hypothesised that MDA5 might have emerged before RIG-I and their domain arrangement evolved independently by domain grafting rather than by a simple gene duplication event [16]. In this study, we took a comparative genomics approach by analysing RIG-I like PRRs in a number of invertebrate and vertebrate genomes, in order to elucidate the origin and evolution of the RIG-I like PRR family. Bioinformatic analysis of functional domains of RIG-I, MDA5 and LGP2 has identified two evolutionary conserved domains in MDA5 and LGP2 which may be critical to the recognition and processing of viral nucleotide PAMPs.

## Results

### Sequence identification

Extensive BLAST analysis of vertebrate genomes or expressed sequence tag databases using known RIG-I protein sequences identified a putative full length RIG-I homologue in Western Clawed Xenopus but not in chicken (Table 1). The putative Xenopus RIG-I is 945 aa in length, sharing 43.6% identity with human RIG-I and contains conserved domains such as a DExD/H domain in the middle region and a helicase domain at the C terminal region. A less homologous CARD domain at the N terminus is also apparent. In the zebrafish genome, a single RIG-I like gene encoding a protein of 628 aa was found in chromosome 23 (Ensemble prediction ID No., ENSDARG00000039785), some 300 aa shorter than the mammalian and amphibian RIG-I proteins. Although it contains a DExD/H domain and a CARD domain at the N terminus, it lacks a classic helicase c (HELICc) domain. It is uncertain whether zebrafish RIG-I is produced as a functional protein since no EST matching the predicted RIG-I exists. In Japanese pufferfish, tetraodon, medaka and stickleback, no RIG-I homologues were identified either in the EST databases or genome databases.

**Table 1:** Sequence information of homologues of RIG-I, MDA5, LGP2, DICER and eIF4A in vertebrates and invertebrates

Gene name	ENSEMBL prediction ID	GenBank GI EST	Protein length	Identity/similarity to human homologue
**RIG-I**				
Human	ENSG00000107201	NM_014314	925	100/100
Xenopus	ENSXETG00000009200		945	43.6/64.2
Zebrafish	ENSDARG00000039785		628	25.9/41.3
				
**MDA5**				
Human	ENSG00000115267	NM_022168	1025	100/100
Chicken	ENSGALG00000011089	XM_422031	1285	47.8/58.8
Xenopus	ENSXETG00000013176		1003	53.3/69.2
Zebrafish	ENSDARG00000018553	XM_689032	1219	34.5/52.4
Stickleback	ENSGACG00000005518		1008	47.1/64.3
Medaka	ENSORLG00000016902		987	47/65.9
Fugu	ENSTRUG00000001413		1000	44.7/63
Tetraodon	ENSTNIG00000016500		1038	42.6/63
				
**LGP2**				
Human	ENSG00000108771	NM_024119	678	100/100
Chicken	ENSGALG00000023821		588	43.6/59.1
Xenopus	ENSXETG00000002302		588	46.8/61.8
Zebrafish	ENSDARG00000070935	XM_001920601	682	40.8/62.5
Stickleback	ENSGACG00000008740		681	47.2/68.9
Medaka	ENSORLG00000003825		673	47.4/66.1
Fugu	ENSTRUG00000015710		671	46.2/64.7
Tetraodon	ENSTNIG00000011713		680	47.7/65.9
				
**DICER**				
Human	ENSG00000100697	NM_177438	1922	100/100
Chicken	ENSGALG00000010999	NM_001040465	1921	92.0/96.0
Xenopus	ENSXETG00000023315		422	18.9/20.6
Zebrafish	ENSDARG00000001129	XM_678382	1975	75.5/84.4
Stickleback	ENSGACG00000020134		1901	75.9/84.5
Medaka	ENSORLG00000011022		1905	76.5/85.2
Fugu	ENSTRUG00000006156		1896	75.8/84.4
Tetraodon	ENSTNIG00000005441		1915	75.8/85.0
Sea urchin DICER		XM_785801	1850	41.0/60.1
*Caenorhabditis elegans *DRH1	F15B10.2	NM_068617	1037	14.2/27.5
*Caenorhabditis elegans *DRH2	C01B10.1		956	14.4/26.1
*Caenorhabditis elegans *DRH3	D2005.5	NM_059760	1119	15.8/28.7
*Caenorhabditis elegans *DCR1	K12H4.8	NM_066360	1845	33.7/53.3
Drosophila DCR1		NM_079729	2249	32.0/50.1
Jewel wasp DICER1		XM_001605237	1917	37.3/59.5
				
**eIF4A**				
Human eIF4A1	ENSG00000161960	BT019879	407	100/100
Chicken eIF4A2		NM_204549	407	90.4/96.3
Xenopus eIF4A	ENSXETG00000020123	NM_001011139	406	94.8/98.5
Zebrafish eIF4A1a	ENSDARG00000040268	NM_198366	406	89.4/96.8
Zebrafish eIF4A1b	ENSDARG00000003032	NM_201510	406	89.2/97.1
Stickleback eIF4A1	ENSGACG00000020072		406	89.7/97.3
Medaka eIF4A1	ENSORLG00000010659		405	89.7/97.3
Fugu eIF4A1	ENSTRUG00000002188		406	78.9/90.4
	ENSTRUG00000017170		408	63.9/81.6
	ENSTRUG00000011113		411	62.6/80.5
Tetraodon eIF4A1	ENSTNIG00000004408		292	55.6/64.9
	ENSTNIG00000017079		352	60.7/73.5
	ENSTNIG00000006865		384	68.1/84.3
	ENSTNIG00000007405		411	69.2/87.1
	ENSTNIG00000004366		316	55.4/66.8
	ENSTNIG00000000303		339	47.7/64.6
Drosophila eIF4A	FBgn0001942	NM_164668	403	72.1/86.7
		NM_164669		
		NM_057247		
		NM_164670		
*Sclerotinia sclerotiorum *eIF4A		XM_001594601	398	74.7/86.5
*Botryotinia fuckeliana *eIF4A		XM_001561331	398	74.4/86.5
				
**Invertebrate RIG-I like genes**				
Sea urchin RIG-I (LOC591972)	NW_001470282	XM_791516	960	26.4/47.8
Sea urchin RIG-I (LOC575036)	NW_001312424	XM_001176480	823	23.8/43.2
Sea urchin RIG-I (LOC767124)	NW_001297703	XM_001203626	303	11.8/19.2
Sea urchin RIG-I (LOC574972)	NW_001312424	XM_775381	968	29.3/50.3
Sea urchin RNA helicase (LOC593153)		XM_792644	996	21.4/41.0
Sea urchin RNA helicase (LOC583008)		XM_001198571	480	16.1/26.4
Sea urchin RIG-I		XM_778463	927	28.7/49.7
Sea urchin RNA helicase (LOC578749)		XM_778903	870	27.2/48.6
Sea urchin RIG-I (LOC582062)		XM_782035	209	10.7/14.5
Sea urchin RIG-I (LOC577076)		XM_777329	1051	19.9/39.7
*Nematostella vectensis *RIG-I/MDA5 like gene 1		XM_001636292 XP_001636342	672	26.3/41.0
*Nematostella vectensis *RIG-I/MDA5 like gene 2		XM_001639190 XP_001639240	689	25.5/41.7

Unlike RIG-I, whose presence in chicken and some fish species is uncertain, the MDA5 homologues can be found throughout vertebrate species including fish, amphibians, birds and mammals (Table 1). MDA5 is encoded by a single copy gene and the putative proteins have comparable length, ranging from 987 aa to 1285 aa (Table 1). In addition to the conserved DExD/H domain and HELICc domain, two tandem CARD domains at the N terminal regions are predicted and are well conserved among vertebrate MDA5 molecules except for the zebrafish MDA5 that lacks a clear CARD domain. The CARD motifs near the N-terminus (referred to as the first CARD) are more diverse than the second CARD motif (Table 2).

**Table 2:** Key structural domains predicted in the Pfam database.

	GenBank Accession number	CARD1	CARD2	Res III	DExD/H	HELICc	TFIIS-C
**RIG-I**							
Human	O95786	1–87	104–191 (0.69)		244–420 (7.1e-14)	655–734 (9.2e-15)	
Xenopus	ENSXETP00000020202		103–189 (0.43)		303–434 (1.6e-10)	723–785 (1.3e-13)	
Zebrafish	ENSDARP00000058175 (partial)		103–186 (3.7)		246–268 (0.019)		
**MDA5**							
Human	Q9BYX4	7–97	115–200 (2.4e-18)	305–493 (3.2e-22)		743–826 (1.1e-20)	
Chicken	XP_422031	37–90 (15)	112–198 (7.8e-06)	298–485 (4.8e-20)		717–800 (5.6e-21)	936–944 (0.51)
Xenopus	ENSXETP00000028841	12–97 (0.073)	142–198 (0.002)	299–487 (7.7e-20)		719–802 (1.1e-21)	938–946 (8.6)
Fugu	ENSTRUP00000003254	10–96 (0.072)	110–196 (3.2e-05)	300–488 (6.4e-15)		711–794 (1.6e-19)	930–939 (1.1)
**LGP2**							
Human	NP_077024				4–178 (1.3e-13)	392–475 (3.3e-21)	
Chicken	ENSGALP00000005315			1–116 (4.3e-05)			521–532 (3.7)
Xenopus	ENSXETP00000004920			1–173 (1.5e-17)		395–478 (7.8e-24)	
Fugu	ENSTRUP00000040150			3–172 (8.5e-19)		396–477 (9.3e-20)	
**DICER**							
Human	NP_803187				45–207 (1.9e-08)	499–556 (7.7e-17)	
Chicken	NP_001035555				45–207 (5.1e-10)	499–556 (7.7e-17)	
Xenopus	ENSXETP00000050382 (partial)				35–209 (6.9e-06)		
Fugu	ENSTRUP00000014961				46–217 (7.3e-06)	499–556 (7.7e-17)	
**eIF4A**							
Human	AAV38682				56–223 (2.5e-63)	291–367 (1.3e-35)	
Chicken	NP_989880				57–224 (6.7e-62)	292–368 (6.5e-36)	
Xenopus	NP_001085314				56–223 (1.1e-62)	291–367 (6.5e-36)	
Fugu	ENSTRUP00000005018				56–223 (5.7e-59)	291–367 (3.6e-36)	

Note: The amino acid position of each domain is listed and the E values against the Pfam database are indicated in parentheses.

LGP2 is an adaptor protein lacking CARD domains but containing a DExD/H domain and a HELICc domain homologous to their corresponding motifs in the RIG-I and MDA5 protein. It competes with RIG-I and MDA5 for the ligands, viral derived RNA PAMPs, but is unable to interact with down stream signalling proteins due to the absence of CARD domains. Thus it acts as a negative regulator of the RIG-I/MDA5 directed antiviral response. LGP2 appears to co-exist with MDA5 in vertebrates as a single copy gene. It is located in a different chromosome to MDA5 in every species analysed. The putative LGP2 proteins from non-mammalian species contain 588–682 aa, much shorter than the RIG-I and MDA5 proteins. The DExD/H domain and the HELICc domain in the LGP2 protein share higher homology with the corresponding regions in MDA5 than those in RIG-I. The LGP2 DExD/H domains are 33.4–55.3% identical to the MDA5 counterparts compared to 22.3–39.8% for the RIG-I proteins. Similarly, 47.6–66.7% identity is seen between the LGP2 HELICc domains and the MDA5 HELICc domains, in contrast to 31.7–48.7% between LGP2 HELICc domains and the RIG-I helicase domains.

Twelve genes coding for RNA helicase proteins homologous to *RIG-I/MDA5/LGP2 *have been reported in a recent survey of the sea urchin genome draft [15]. Some of the deduced proteins contain CARD domains in addition to DExD/H and HELICc domains. Using the human MDA5 protein sequence as a bait, a partial homologue sequence was obtained from the sea anemone *Nematostella vectensis *genome database ,  This partial sequence was then used to search the NCBI database and two contigs (NEMVEDRAFT_v1g95706 and NEMVEDRAFT_v1g87071) were retrieved, which encoded two putative RIG-I/MDA5/LGP2 homologues. The putative proteins are 672 aa and 689 aa in length, similar to that of LGP2. Further prediction of functional motifs revealed the presence of a DExD/H domain and a HELICc domain but not the N terminal CARD domain. The proteins share 17.4–26.3 identity with RIG-I, 21.7–32.4% with MDA5 and 25.3–36.3% with LGP2.

### Gene synteny analysis

**Figure 1:**
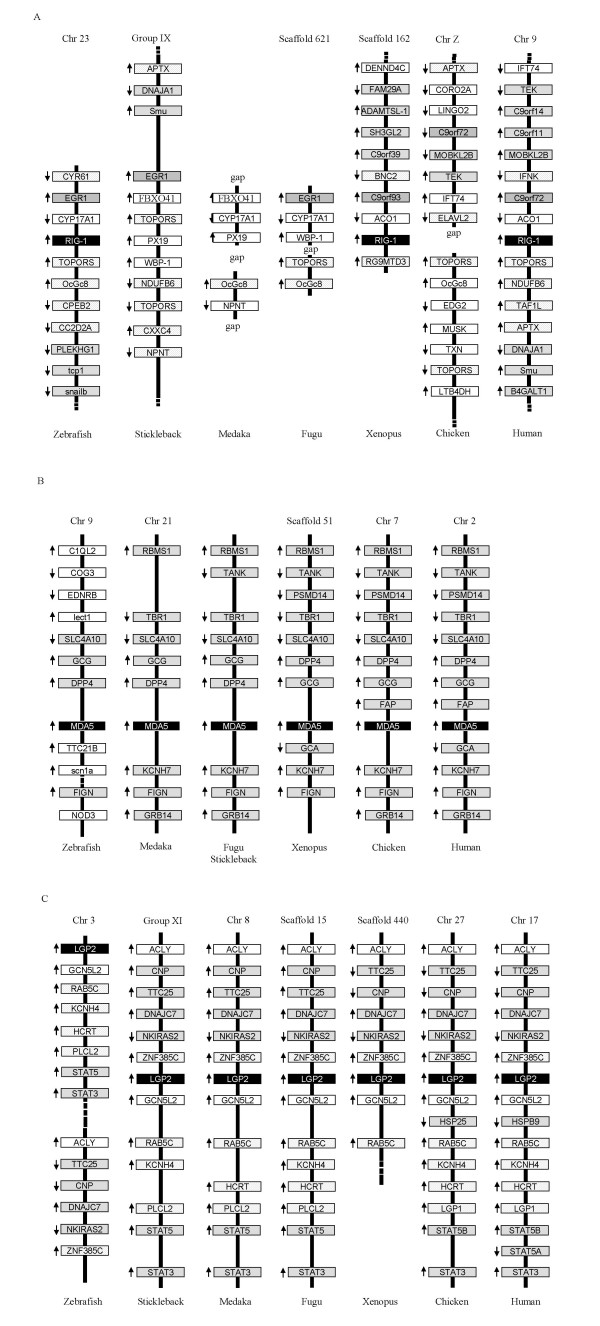
**Comparative analysis of gene synteny of RIG-I (A), MDA5 (B) and LGP2 (C) in vertebrate genomes**. The gene symbols are used according to the human genome map (Build 36.3, ).

To gain an insight into whether the genes surrounding *RIG-I, MDA5 and LGP2 *are evolutionary conserved, we analysed draft genome sequences of invertebrates, fish, amphibians, birds and humans. The *RIG-I *locus was identifiable in Xenopus where the genes neighbouring *RIG-I *were different to those in humans except for the *ACO1 *gene (Fig. 1A). Since the upstream region of the *RIG-I *locus was not available in the Xenopus genome database, whether *RIG-I *clustered with *TOPORS *remains to be determined. In zebrafish, the *RIG-I *gene was immediately downstream of the *TOPORS *gene, which is adjacent to the *RIG-I *gene in humans. In contrast to the observation in zebrafish, the stickleback genome appears to lack *RIG-I*, which is not due to poor genome analysis since the sequence of the *RIG-I *locus is of good quality. In Fugu, medaka and chicken, the *RIG-I *gene could not be identified but was hindered by incomplete sequence data.

The gene synteny of *MDA5/LGP2 *is well conserved in vertebrates, from fish to humans (Fig. 1B and 1C). Eight genes surrounding *MDA5 *in stickleback, Fugu and medaka appear in the genomes of Xenopus, chicken and humans, in the same order and the same transcriptional orientation. Less conservation of gene synteny was noted in the zebrafish genome where only 4 conserved neighbouring genes were present in the *MDA5 *locus. Similarly, the gene composition and arrangement in the *LGP2 *gene locus shows remarkable conservation during vertebrate evolution.

**Figure 2:**
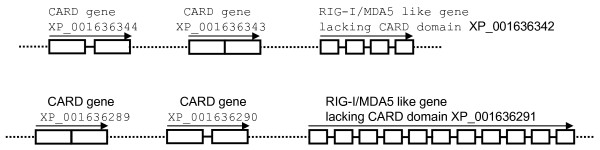
**Genomic location of predicted RIG-I/MDA5 homologues in sea anemone *Nematostella vectensis***. Exons and gene transcriptional orientation are indicated by blank boxes and arrows respectively. The accession numbers of predicted genes are given.

In invertebrates, two loci containing prototype homologues of *RIG-I/MDA5 *were found downstream of two independent genes coding for two CARD like molecules in *Nematostella vectensis *(Fig. 2). Some 12 *RIG-I/MDA *like genes have also been predicted from the purple sea urchin genome [15]. Whilst it was possible to find appropriate contigs containing *RIG-I/MDA5 *like genes from the sea urchin genome project, it was not possible to assess gene synteny due to a lack of genome assembly.

### Phylogenetic analysis

**Figure 3:**
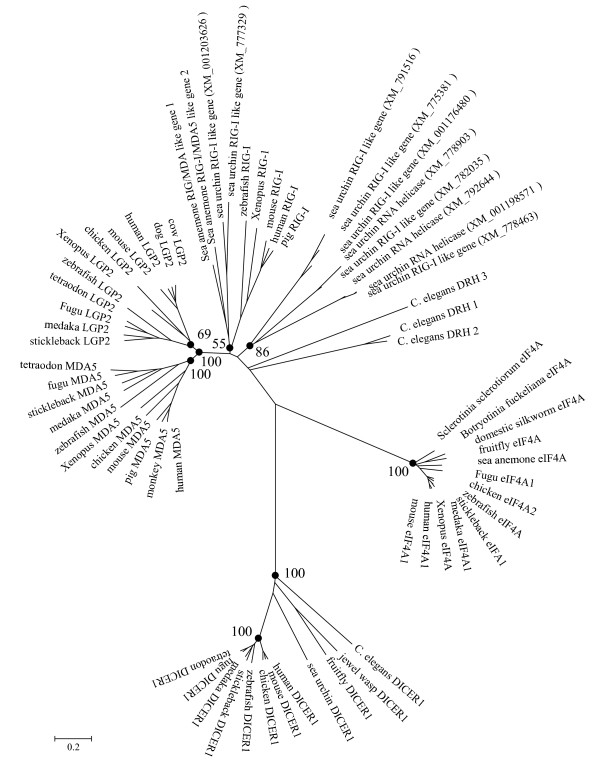
**Phylogenetic tree analysis of DExD/H box RNA helicases**. Multiple alignment of the full length protein sequences of known or predicted RIG-I, MDA5, LGP2, eIF4A and DICER was generated by CLUSTALW  and used for construction of a phylogenetic tree using the neighbour-joining method within the Mega3.1 programme. The bootstrap values of the branches were obtained by testing the tree 10,000 times and values over 50% percent marked. The sequences used for tree construction are listed in Table 1. Others are as follows: monkey MDA5, NP_001040588; mouse MDA5, EDL26991; pig_MDA5, NP_001093664; mouse LGP2, NP_084426; cow LGP2, NP_001015545; dog LGP2, XP_860567; mouse RIG-I, Q6Q899; pig RIG-I, Q9GLV6; mouse DICER1, EDL18787.

RIG-I, MDA5 and LGP2 are believed to bind RNA molecules through helicase-C domains. Closely related proteins containing a RNA binding helicase-C domain and a DExD/H helicase domain are the double-stranded RNA-specific endoribonuclease (DICER) and elongation initiation factor (eIF) 4A. DICER belongs to the RNase III family that cleaves double-stranded RNA (dsRNA) into short double-stranded RNA fragments, called small interfering RNA, that is required for specific cleavage of complementary viral RNAs, whilst eIF4A mainly participates in translation and other processes such as pre-mRNA splicing and ribosome biogenesis. Since homology analysis of the RIG-I and MDA5 proteins in the databases using the BLASTP programme gave top scores to DICER and eIF4A in addition to the RIG-I, MDA5 and LGP2 proteins, we included DICER and eIF4A in the phylogenetic tree analysis using the neighbour joining method within the Mega4 programme. As shown in Fig. 3, DICER and eIF4A formed two distinct groups with a long evolutionary distance to the branch that evolved into invertebrate DICER like helicase (DRH), RIG-I, MDA5 and LGP2. Apparently, DICERs are more distantly related to the ancestor of RIG-I, MDA5 and LGP2 than eIF4A. Comparing to DICERs and eIF4As, *C. elegans *DRHs were located close to some of the sea urchin RIG-I/MDA like proteins, the prototype of the vertebrate counterparts. Within the RIG-I branch are two of the RIG-I like homologues from *Nematostella vectensis*, two from the sea urchin (LOC767124 and LOC577076), and predicted or known RIG-I molecules from zebrafish, Xenopus, and mammals, suggesting RIG-I may have emerged in invertebrates. The fact that RIG-I homologues were absent in most fish species suggests that the *RIG-I *gene may have been lost in some fish lineages. The tree also indicates that LGP2 and MDA5 proteins from vertebrate species form two clear groups closely neighbouring each other, suggesting they possibly diverged from a common ancestor that originated from a RIG-I like molecule in invertebrates or early vertebrates. In addition, two phylogenetic trees were constructed using helicase C domains and DExD/H domains and a similar grouping of the major branches was observed (data not shown).

### Domain analysis

**Figure 4:**
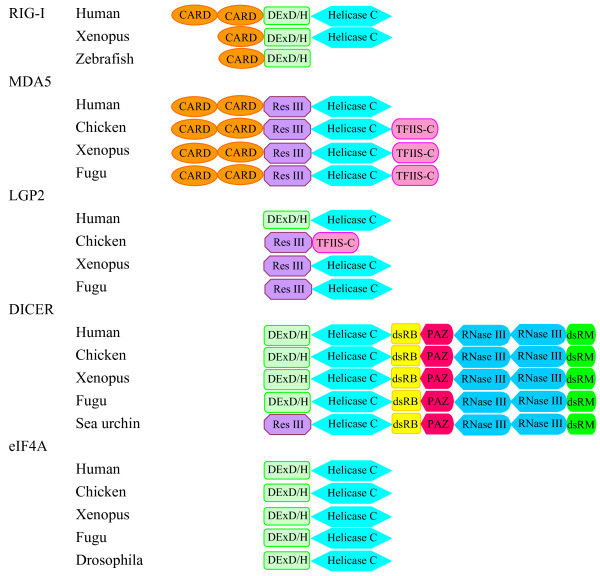
**Schematic of key functional domains of RIG-I, MDA5, LGP2, DICER and eIF4A predicted by the Pfam HMM programme**.

The putative domains were analysed in the Pfam database using a cut-off E-value of 10.0 and selected domains listed in Table 2. A comprison of the domains is shown in Fig. 4. A classical helicase C domain is present in all 5 types of DExD/H helicases, including RIG-I, MDA5, LGP2, DICER and eIF4A. The DExD/H box responsible for ATP binding and hydrolysis was detected in the middle region of RIG-I, DICER and eIF4A by the Pfam HMM search. Similarly, the corresponding region in MDA5 and LGP2 was detected as a conserved restriction domain of bacterial type III restriction enzymes (Res III) (E-values between 4.3e-15 and 3.2e-22), sharing some degree of homology with the RIG-I DExD/H box. Two CARD domains were predicted in the N terminal regions of RIG-I and MDA5 except for Xenopus and zebrafish RIG-I, which contained a single CARD domain corresponding to the second CARD domain of the human molecule (CARD2). In general, the N-terminal CARD domain (CARD1) was less conserved than the CARD2 domain. The E-value of CARD2 in RIG-I ranges between 0.43 and 3.7, significantly higher than that in MDA5 (0.002–2.4e-18), indicating significant divergence of the CARD domains in different vertebrate groups. In addition, a fragment at the C-terminal region in most MDA5 proteins of chicken, Xenopus and fish, contained a putative domain with moderate E-values (0.51–8.60) to the 4 cysteine (C4) type transcription factor (TF) IIS central domain. The C terminal region of human RIG-I comprised a region distantly related to the C4 type zinc finger domain, which was shown to bind to dsRNA and 5'ppp viral RNA with the involvement of zinc ion [12,17]. The Pfam HMM analysis failed to identify this C4 TFIIS domain in RIG-I possibly due to low homology.

**Figure 5:**
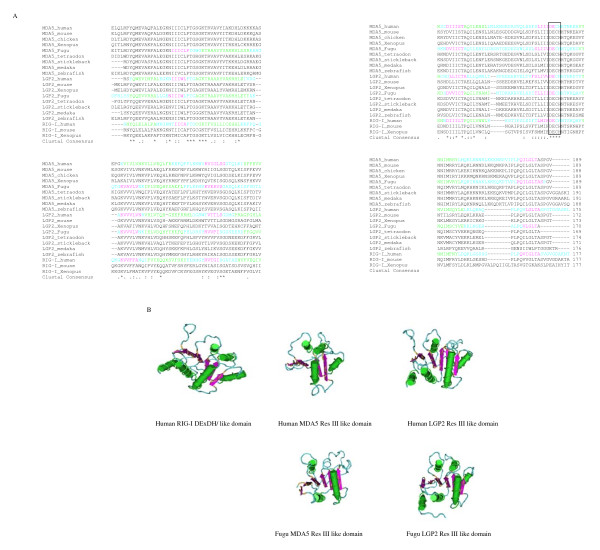
**Multiple alignment (A) and structural modelling (B) of Res III**. The domain sequences were predicted by the Pfam HMM programme and aligned using the CLUSTALW programme. The 3-dimensional structures of the domains were generated using the 3D-jigsaw comparative modelling programme and the VAST search programme and visualised by the Cn3D programme (Version 4.1). Identical (*) and similar (:, .) residues are shown below the alignment. The conserved signature "DECH" is boxed. Amino acid position of the domain position is listed in Table 2. Predicted α-helices (green), β-strands (purple or dark blue) and loops (light blue) are marked in both the alignment and the 3D structures respectively.

**Figure 6:**
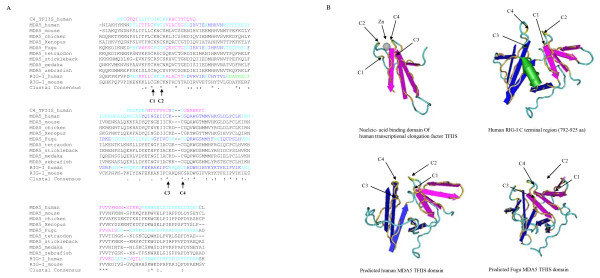
**Multiple alignment (A) and structural modelling (B) of TFIIS domains**. The domain sequences were predicted by the Pfam HMM programme and aligned using the CLUSTALW programme. The 3-dimensional structures of the domains were generated using the 3D-jigsaw comparative modelling programme and the VAST search programme and visualised by the Cn3D programme (Version 4.1). Identical (*) and similar (:, .) residues are shown below the alignment. Cysteines (C1–4) involved in zinc (Zn) binding are indicated by arrows in the alignment and the 3D structures. Amino acid position of the domain position is listed in Table 2. Predicted α-helices (green), β-strands (purple or dark blue) and loops (light blue) are marked in both the alignment and the 3D structures respectively.

The domain sequences were further analysed by multiple alignment and their 3 dimensional structures modelled. It is apparent that the DExD/H and Res III domains comprised a well conserved DECH motif (Fig. 5A). The overall structures of DExD/H boxes and Res III domains were similar, with β sheets sandwiched by α-helices on each side (Fig. 5B). Six β strands were arranged in the same orientation in the human RIG-I DExD/H box and MDA5/LGP2 Res III domains except for the human MDA5 Res III domain, where 4 β strands were present. Conversely, numbers of predicted α-helices varied significantly among domains. Although homology analysis showed low sequence similarities between the MDA5 TFIIS domain and the C4 type zinc finger nucleotide binding motif within the human TFIIS central domain, 3D modelling displayed significant structural similarities (Fig. 6). Our modelling data indicated the C terminal region of RIG-I/MDA5 proteins across vertebrates possessed a conserved C4 type zinc finger nucleotide binding motif, in agreement with the studies in humans where a putative domain distantly related to the C4 type zinc finger protein was shown to bind to viral nucleotide PAMPs [12]. Remarkably, the 4 cysteines involved in capturing zinc ion were in close physical contact, forming a conserved pocket on the surface of all the domains analysed. However, arrangement of the cysteines differed in the human TFIIS-C domain and RIG-I/MDA5 domains (Fig. 6). In addition to the C4 type zinc finger nucleotide binding motif, the RIG-I C terminal region and the MDA5 TFIIS domain contained another noticeable β strand structure which appeared to support the C4 type pocket. In the human RIG-I, a single α helix was also detected.

## Discussion

The RIG-I like helicase family members have recently been reported to play pivotal roles in recognising viral nucleotides in mammals. In this report, the RIG-like homologues have been identified *in silico *in the nucleotide databases of invertebrates and vertebrates and their evolutionary origin discussed.

Double stranded RNA is the genetic component of viruses with double stranded genomes and part of a single stranded RNA with secondary structures. It can be generated during viral replication and RNA metabolism. This nature of dsRNA makes it the prime target for host PRRs. Classical double stranded RNA binding domains are often used by some cytosolic PRRs, such as PKRs, as the detectors to sense viral presence. DICER proteins also contain two dsRNA binding domains (dsRBDs) for capturing dsRNA molecules. In the present study, a zinc finger domain similar to that of transcription factor (TF) IIS has been found in MDA5 but not in LGP2, with moderate homology to the RIG-I C terminal region. Furthermore, a well conserved type III restriction enzyme domain responsible for restriction in prokaryotic organisms is identified in the middle of both MDA5 and the N terminal region of LGP2. This domain was not detected in RIG-I molecules by the Pfam HMM programme although it shared some degree of homology. We speculate that these two domains may serve as potential binding domains to interact with viral PAMPs.

One striking finding is that a well conserved restriction enzyme III (Res III) domain is predicted in all MDA5 and LGP2 proteins (except human LGP2). The Res III domain is structurally similar to the DExD/H domain. Restriction enzymes are important components of prokaryotic DNA restriction-modification mechanisms in defence against foreign DNA [18]. They function in combination with one or two modification enzymes (DNA-methyltransferases) that protect the cell's own DNA from cleavage by the restriction enzymes. Restriction enzymes consist of four types depending on their recognition sequences and location of cleavage sites. Type III enzymes recognize short 5–6 bp long asymmetric DNA sequences and cleave 25–27 bp downstream to generate short, single-stranded 5' overhang ends. Type III enzymes contain two functional subunits Res (restriction) and Mod (modification), specifically for DNA cleavage of unmethylated double stranded foreign DNA (Res unit) and protection of self DNA from damage by methylation (Mod unit), respectively. Classic strand separation helicase activities have not been detected for type III restriction enzymes [19]. The Res III domain predicted in MDA5 and LGP2 have significant homology with bacterial Res III domains and multiple alignment reveals significant conservation (Fig. 5A). MDA5/LGP2 are also similar to the RNase III domains in the RNA endonuclease DICER and DICER like helicases which process dsRNA into 21–23 nt 3' overhang small RNAs, with 2 nt protrusions, and ATP-binding domains in bacterial and yeast DNA helicases [20,21]. Integrated nuclease domains with excision activities are seen in the DICER proteins where two ribonuclease III domains cut double stranded RNAs, releasing 2 nt 3' end overhang 21–23 nt RNA molecules which are essential for specific cleavage of viral RNAs [20,22].

Another putative important domain, a zinc finger motif similar to that of transcription factor (TF) IIS, was identified by homology analysis in the Pfam database. The zinc finger motif can bind a range of targets including DNAs, RNAs, proteins and even lipids. It is known that the zinc finger motif at the C-terminus of the TFIIS is essential for RNA binding and processing [23]. Integrated TFIIS zinc ribbon C-terminal domains are also found in some viral proteins [24,25]. The TFIIS motif located near the C terminus of the RIG-I/MDA5 proteins was detectable in the Pfam database although the E value (0.51–8.6) is moderate (Table 2). Structural modelling confirmed remarkable conservation of a C4 type zinc finger pocket and a β-strand structure compared to the C4 type zinc finger nucleic acid binding domain in the human TFIIS. Furthermore, a β-strand motif is also present within this domain in addition to the C4 type β-strand zinc finger structure. Whether it is involved in recognition of viral RNA PAMPs remains to be determined. A recent study has demonstrated that a C terminal domain in human RIG-I (792–925 aa) was involved in binding dsRNA or 5'ppp RNA, which was confirmed by magnetic resonance and X-ray crystallography [12,13]. This region was also shown to suppress RIG-I signalling [8]. Thus it is possible that viruses could interfere with this host recognition system by their own TFIIS-C containing proteins.

**Figure 7:**
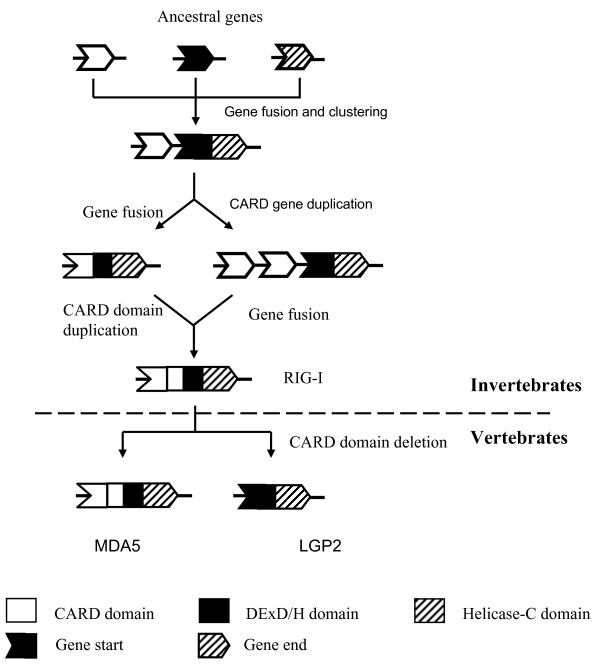
**Hypothetical evolutionary origin of *RIG-I*, *MDA5 *and *LGP2***.

The origin and evolution of *RIG-I, MDA5 and LGP2 *were analysed in this study. Our data suggest they evolved from common invertebrate ancestors encoding distinct core domains (Fig. 7), which was supported by the presence of the *RIG-I *like genes in sea anemone *Nematostella vectensis *and sea urchin genomes [15]. Tandem clustering of two CARD genes with the *RIG-I *like genes without CARD domains in the *Nematostella vectensis *genome provides a strong clue as to how *RIG-I, MDA5 *and *LGP2 *could have evolved during evolution through gene fusion, domain duplication and domain deletion (Fig. 7), supporting recent analysis suggesting that CARD1 could have been grafted independently rather than duplicated from CARD2 during evolution [16]. In Deuterostome invertebrates, the *RIG-I/MDA5 *like genes appear to have expanded enormously, as seen in the sea urchin, with some differentiating into molecules with a closer phylogenetic relationship to the vertebrate *RIG-*I molecules (Fig. 3). We speculate that RIG-I emerged earlier than MDA5/LGP2 since vertebrate RIG-Is grouped with the invertebrate progenitors rather than the MDA5 and LGP2 proteins which are present uniquely in vertebrates, in stark contrast with the evolutionary model proposed by Sarkar et al [16], where LGP2 preceded both MDA5 and RIG-I in evolution. Moreover, the phylogenetic tree constructed in the present study shows that MDA5 has a closer relationship with LGP2 rather than RIG-I, suggesting MDA5 and LGP2 originated from a more recent gene duplication event, unlike the phylogenetic results obtained by Sarker et al [16]. If MDA5/LGP2 did diverge from RIG-I more recently, the order of their appearance is not clear. Also, it is uncertain from the present study why RIG-I was not found in all teleost fish. Although a putative gene coding for a partial RIG-I is predicted in the zebrafish genome, with conserved gene synteny to the Xenopus and human *RIG-I *locus (Fig. 1A), it is absent in the other fish genomes. Poor quality of the genome sequences makes conclusions difficult but as no *RIG-I *sequences were found in the vast number of fish EST sequences, perhaps functional *RIG-I *genes have been lost in some teleost fish species.

## Conclusion

The RIG-I/MDA5/LGP2 system is an ancient antiviral system well conserved in vertebrates. Our data suggest that these helicase PRRs have evolved from an ancient progenitor originated from genes coding for individual functional domains and expanded by multiple evolutionary events leading to gene and/or domain gain and loss. The present study provides important clues for further elucidation of RIG-I/MDA5 mediated antiviral defence in vertebrates.

## Methods

### Database mining

To identify *MDA5*, *LGP2 *and *RIG-I *genes in the available teleost genomes, the tblastn search using the human MDA5, LGP2 and RIG-I protein sequences as baits was performed against the genomes of zebrafish (*Danio rerio*), pufferfish (*Takifugu rubripes *and *Tetraodon nigroviridis*), medaka (*Oryzias latipes*), stickleback (*Gasterosteus aculeatus*), Western Clawed Xenopus (*Xenopus tropicalis*) and Chicken (*Gallus gallus*) in the Ensembl database . The obtained sequences were reciprocally searched against the other genomes to further verify their identity. The translated proteins from predicted transcripts were verified by BLASTP in the NCBI non-redundant protein sequence database and the SWISSPROT protein database . In addition, known *MDA5*, *LGP2 *and *RIG-I *genes were retrieved from the NCBI database for analysis.

For gene synteny analyis, human *MDA-5*, *LGP2 *and *RIG-I *were used as anchor sites. Orthologous comparisons of the genes in the regions of approximately 1 to 10 mb (million base pairs) flanking the human (NCBI 36) anchor site with medaka (HdrR), zebrafish (Zv7), stickleback (BROAD S1), pufferfish (FUGU 4.0, TETRAODON 7), Western Clawed Xenopus (JGI 4.1) or chicken (WASHUC2) genome were done within the Ensembl genome browser using the GeneView and MultiContigView options. Manual annotation of orthologous genes was also performed using FGENESH+ to predict structures based on homology with human genes: "fish" specific parameters were applied in this program.

### Sequence analysis

The conserved domains were predicted using software at the ExPASy Molecular Biology Server . Caspase recruitment domain, DExD/H box helicase, Type III restriction enzyme and helicase conserved C-terminal domains were predicted by a Pfam HMM search with a cutoff value of 10.0. The full-length amino acid sequences and the conserved functional domains were used in phylogenetic tree analysis. Multiple protein sequence alignments were performed using the ClustalW programme (version 1.83) [26]. A phylogenetic tree was constructed using the neighbour-joining method within the MEGA (4.0) package [27]. Data were analyzed using Poisson correction, and gaps were removed by pairwise deletion. The topological stability of the neighbour-joining trees was evaluated by 10,000 bootstrap replications. The three dimensional (3D) structures were predicted using the 3D JIGSAW protein comparative modelling programme [28] and compared to those in the MMDB/PDB database by VAST search analysis . The 3D structural images were displayed by the Cn3D programme (version 4.1).

## Authors' contributions

JZ initiated the study, performed bioinformatic and phylogenetic analysis, and drafted the manuscript. MXC made substantial contributions to bioinformatic analysis. PN and CJS advised on data analysis and interpretation. CJS contributed significantly in editing the manuscript. All authors read and approved the final manuscript.
